# Proposal of a Geometric Calibration Method Using Sparse Recovery to Remove Linear Array Push-Broom Sensor Bias

**DOI:** 10.3390/s19184003

**Published:** 2019-09-16

**Authors:** Jun Chen, Zhichao Sha, Jungang Yang, Wei An

**Affiliations:** College of Electronic Science, National University of Defense Technology, Changsha 410073, China; shazhichao163@163.com (Z.S.); yangjungang@nudt.edu.cn (J.Y.); anwei@nudt.edu.cn (W.A.)

**Keywords:** geometric calibration, long- and short-period errors, equivalent bias angles, sparse recovery, linear array push-broom sensor

## Abstract

The rational function model (RFM) is widely used in the most advanced Earth observation satellites, replacing the rigorous imaging model. The RFM method achieves the desired calibration performance when image distortion is caused by long-period errors. However, the calibration performance of the RFM method deteriorates when short-period errors—such as attitude jitter error—are present, and the insufficient and uneven ground control points (GCPs) can also lower the calibration precision of the RFM method. Hence, this paper proposes a geometric calibration method using sparse recovery to remove the linear array push-broom sensor bias. The most important issue regarding this method is that the errors related to the imaging process are approximated to the equivalent bias angles. By using the sparse recovery method, the number and distribution of GCPs needed are greatly reduced. Meanwhile, the proposed method effectively removes short-period errors by recognizing periodic wavy patterns in the first step of the process. The image data from Earth Observing 1 (EO-1) and the Advanced Land Observing Satellite (ALOS) are used as experimental data for the verification of the calibration performance of the proposed method. The experimental results indicate that the proposed method is effective for the sensor calibration of both satellites.

## 1. Introduction

With the accelerating development of technical aeronautics and space exploration, many countries have launched advanced Earth observation satellites in recent years. The linear array push-broom sensor is the payload which has been carried by the most satellites. This significant quantity of Earth observation has had a great impact on overall production and human life. However, the imaging process of sensors is easily influenced by a variety of errors, including thermal deformation error, optical distortion error, satellite position, attitude measurement errors, and so on [1,2]. Commonly, the raw images contain many image distortions and cannot be directly applied to the processing of subsequent applications. Typically, errors which result from image distortion can be divided into two sources: long-period errors, including assembling error, optical distortion error, and thermal distortion error, which are constant, non-varying, or slowly varying [3]; and short-period errors, including satellite position, attitude measurement errors, and attitude jitter error. If these errors are insufficiently characterized or uncorrected, significant distortion of the raw image can result. Therefore, it is necessary to complete geometric calibration of the sensor to eliminate image distortion before raw image application.

The imaging process involves projecting a point on the surface of the Earth toward the sensor’s focal plane [4]. The imaging models are used to describe this imaging process consist of two types [5]. The first type is the rigorous imaging model, which involves a set of coordinate transformations. Many rigorous image models have been proposed by different scholars for a variety of sensors in previous research [1,2,6,7]. However, most imagery vendors often do not provide details of advanced Earth observation satellites, such as the precise parameters and work mode of sensor or the satellite orbit, to the user. Therefore, this type of model has many limitations in practical use. The other type is the generalized imaging model. There are a lot of generalized imaging models, with the main ones being the rational function model (RFM), the direct linear transformation model, and the polynomial model. The RFM is widely applied in sensor calibration due to its simple form and high precision compared to the other models [5]. However, the RFM has strict requirements regarding the distribution and number of ground control points (GCPs) in each frame, and it also needs to calculate the coefficients frame-by-frame. The RFM method effectively calibrates image distortion caused by the long-period errors—such as thermal distortion error, optical distortion error, and assembling error—but the calibration performance of the RFM method deteriorates in the presence of short-period errors, such as attitude jitter [8]. The majority of advanced Earth observation satellites undergo attitude jitter, which results from attitude control operations, the dynamic structure, and so on [9]. Referring to the previous studies [8,9], we found three classic ways to handle satellite attitude jitter. The first way is to apply an advanced hardware device, such as an angular displacement sensor, to achieve high-resolution measurement data. However, this is infeasible for many satellites due to restrictions regarding the economy and technology. The second way is to estimate attitude jitter by using multispectral parallax images. This method is unsuited for the estimation of attitude jitter of satellites which are not equipped with multispectral sensors. The third way is to use high precision GCPs in scenes; the effectiveness of this method is decided by the distribution and number of GCPs [8]. However, it is difficult to extract many GCPs with high precision in each scene, especially if water or cloud coverage exists in the scene.

In order to solve the above-mentioned problems, this paper puts forward a geometric calibration method by using sparse recovery to remove linear array push-broom sensor bias. The errors relating to the imaging process are approximated to the equivalent bias angles [10], and the equivalent bias angles of the image sequence are recovered by compressive sensing in this method. Hence, the traditional problem regarding error-solving in the sensor calibration process is transformed into a new problem regarding signal recovery in this paper.

## 2. Sensor Geometric Calibration Model

### 2.1. Sensor Geometric Calibration Modeling

**Figure 1:**
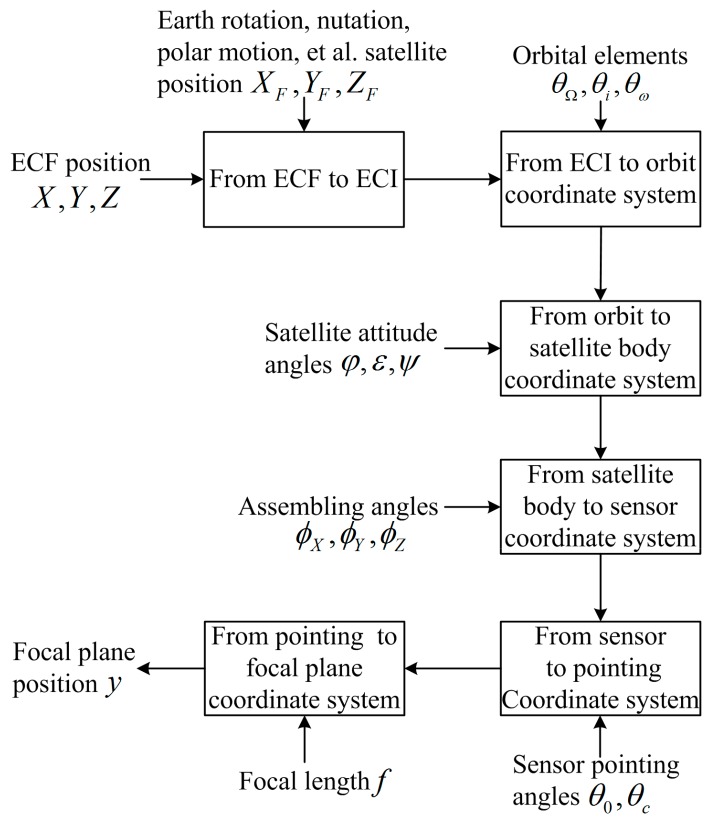
Basic flow diagram of the imaging process for the linear array push-broom sensor.

Over the past decade, the linear array push-broom sensor has been used as a main payload assembled by the most advanced Earth observation satellites. The imaging process of the linear array push-broom sensor projects a point on the surface of Earth, such as a GCP, to the sensor’s focal plane [4]. The basic flow diagram of this process is shown in Figure 1. The rigorous imaging model for the linear array push-broom sensor is presented as follows [6,7,11].

(1) $$\left[\begin{array}{c} X-X_{F}\\ Y-Y_{F}\\ Z-Z_{F} \end{array}\right]=m\times \;R_{ECI}^{ECF}\times \;R_{orb}^{ECI}(\theta_{\Omega },\theta_{i},\theta_{\omega })\times R_{orb}^{body}(\varphi ,\varepsilon ,\psi )\;\times \;R_{body}^{sen}(\phi_{X},\phi_{Y},\phi_{Z})\times \;M_{mir}(\theta_{0},\theta_{c})\times \;\left[\begin{array}{c} 0\\ y\\ -f \end{array}\right]$$

where $\left(X,Y,Z\right)$ represents the projective position in the Earth-centered fixed (ECF) coordinate system, y represents the image column position in the focal plane coordinate system, $\left(X_{F},Y_{F},Z_{F}\right)$ represents the satellite position in the ECF, m represents a scale factor, f represents the focal length, $R_{ECI}^{ECF}$ denotes the rotation from the Earth-centered inertial (ECI) to the ECF coordinate system, $\theta_{\Omega },\theta_{i},\theta_{\omega }$ represent the orbital elements of the satellite (right ascension of the ascending node, inclination, and true perigee angle), $R_{orb}^{ECI}$ denotes the orbital elements that give the rotation from the satellite orbit to the ECI coordinate system, φ,ε,ψ represent the Euler angles of the satellite attitude, $R_{orb}^{body}$ denotes the satellite attitude angles that give the rotation from the satellite body to the satellite orbit coordinate system, $\phi_{X},\phi_{Y},\phi_{Z}$ represent the assembling angles of the sensor, $R_{body}^{sen}$ denotes the assembling angles that give the rotation from the sensor to the satellite body coordinate system, $\theta_{0},\theta_{c}$ represent the sensor pointing angles (the mirror assembling angle and the mirror rotation angle), and $M_{mir}$ denotes the sensor pointing angles that give the rotation from the pointing to the sensor coordinate systems.

Compared with the true values, the imaging parameters usually solve some undistinguished errors in the imaging process. The long-period errors, such as thermal distortion error, optical distortion error, assembling error, and the short-period errors—such as satellite orbit error and attitude error—of the imaging process are approximated to the equivalent bias angles. The sensor geometric calibration model is presented as [1,2,10]

(2) $$\left[\begin{array}{c} X-X_{F}\\ Y-Y_{F}\\ Z-Z_{F} \end{array}\right]=m\times \;R_{Eq}(\alpha ,\beta ,\theta )\times \;R_{ECI}^{ECF}\times \;R_{orb}^{ECI}\times \;R_{orb}^{body}\times \;R_{body}^{sen}\times \;M_{mir}\times \;\left[\begin{array}{c} 0\\ y\\ -f \end{array}\right]$$

where α,β,θ represent the equivalent bias angles and $R_{Eq}(\alpha ,\beta ,\theta )$ denotes the equivalent rotation matrix

(3) $$R_{Eq}(\alpha ,\beta ,\theta )=\left(\begin{array}{ccc} 1 & 0 & 0\\ 0 & \cos\alpha & \sin\alpha \\ 0 & -\sin\alpha & \cos\alpha \end{array}\right)\left(\begin{array}{ccc} \cos\beta & 0 & -\sin\beta \\ 0 & 1 & 0\\ \sin\beta & 0 & \cos\beta \end{array}\right)\left(\begin{array}{ccc} \cos\theta & -\sin\theta & 0\\ \sin\theta & \cos\theta & 0\\ 0 & 0 & 1 \end{array}\right)$$

### 2.2. Error Modeling

All on-orbit satellites suffer from an environment that varies cyclically between cooling and heating due to the circular orbit around Earth. The thermal distortion error resulting from the satellite in the Earth’s orbit causing a solar eclipse is the biggest error in the imaging process [12]. The period of this cycle is the same as the orbit period of the satellite. Thermal distortion errors present in three directions, with the error in the direction toward the sun being the largest. The optical distortion error and assembling error are constant bias errors or long-period variation errors [4]. Attitude jitter is also a primary source of decreasing sensor performance. The satellite position and attitude measurement errors, which are a type of Gaussian error, can be effectively removed by using compressive sensing, therefore, they are not included in the bias angle model. According to the characteristics of these errors, the equivalent bias angles can be described by the constant errors and a set of period components varying in sinusoidal waveform, demonstrated as [4,12]

(4) $$\left\{\begin{array}{c} \alpha (t)=\varsigma_{\alpha }+\sum_{i}^{n}A_{\alpha i}\sin(2\pi f_{\alpha i}t+\zeta_{\alpha i})\\ \beta (t)=\varsigma_{\beta }+\sum_{i}^{n}A_{\beta i}\sin(2\pi f_{\beta i}t+\zeta_{\beta i})\\ \theta (t)=\varsigma_{\theta }+\sum_{i}^{n}A_{\theta i}\sin(2\pi f_{\theta i}t+\zeta_{\theta i}) \end{array}\right.$$

where $\varsigma_{\alpha },\varsigma_{\beta },\varsigma_{\theta }$ represent the constant errors, $A_{\alpha i},A_{\beta i},A_{\theta i}$ represent the amplitudes of the period components, $f_{\alpha i},f_{\beta i},f_{\theta i}$ represent the frequencies of the period components, and $\zeta_{\alpha i},\zeta_{\beta i},\zeta_{\theta i}$ represent the phases of the period components.

## 3. Geometric Calibration by Using Sparse Recovery

### 3.1. Compressive Sensing

As a new theory in signal processing, compressive sensing was proposed in 2004 and rapidly developed in the following years. The core idea of this theory mainly involves two aspects. The first is the sparsity of the signal, which means that the majority of the elements are have a value of zero or are otherwise very small [13]. The other is sampling irrelevance, in other words, the measurement matrix required to meet the restricted isometry property (RIP) [14]. The traditional Nyquist–Shannon sampling theorem indicated that the sampling frequency must exceed the Nyquist sampling frequency to restore the signal without distortion. However, the sparse signal is exactly recovered by compressive sensing from its incomplete measurements far below the Nyquist sampling frequency [13]. In practical problems, the majority of signals are not initially sparse. Fortunately, these signals can be converted into sparse signals using sparse transformation. Sparse transformation can be done by discrete Fourier transformation (DFT), discrete cosine transformation, and wavelet transformation. Hence, these signals are recovered by compressive sensing. Because of its incomparable advantage in handling large-scale compressible data, compressive sensing is widely used in the fields of radio communication, array signal processing, medical imaging, and so on.

### 3.2. Procedure of Proposed Method

**Figure 2:**
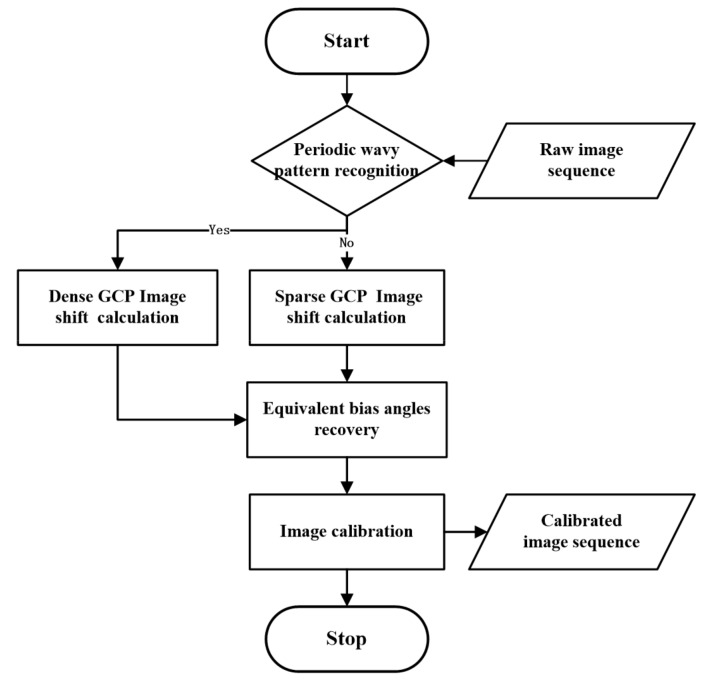
Flowchart of the proposed method.

The geometric calibration method consists of five primary steps: (a) periodic wavy pattern recognition; (b) sparse GCP image shift calculation; (c) dense GCP image shift calculation; (d) equivalent bias angles recovery; and (e) image calibration. The procedure of the proposed method is shown in Figure 2.

#### 3.2.1. Periodic Wavy Pattern Recognition

The first step is to recognize the periodic wavy pattern of the raw image sequence frame-by-frame. Once the raw image exists with periodic wavy patterns, which are caused by short-period error, such as attitude jitter error, the subsequent process of Step 3 is necessary for the frame. There are three processes that need to be completed for periodic wavy pattern recognition. First, a line or edge detection algorithm is used to extract the road, dike, and coastline from the raw images. Then, the smoothness of the extracted lines or edges is extracted. Finally, the existence of periodic wavy patterns in the images is determined. The details of this step can be seen in [15].

#### 3.2.2. Sparse GCP Image Shift Calculation

There are three main processes in this step. First, a handful of images with good imaging conditions are randomly selected from the raw image sequence, excluding images with periodic wavy patterns, as the measurement scenes. Then, a small number of GCPs with high precision from each measurement scene are extracted and matched. The sampling interval of GCPs is set to larger, and these GCPs are defined as sparse GCPs in this paper. The latitude and longitude ranges of each measurement scene are calculated to determine whether there are GCPs in each measurement scene; the templates of GCPs used in this GCPs extraction process are obtained from the Shuttle Radar Topography Mission data. The details of this process are seen in [16]. Finally, we calculate the ideal position of the GCPs in the focal plane coordinates using Equation (2), and the equivalent bias angles are set to zero in this process. The real position of the GCPs are measured from the raw images and the image shift of sparse GCPs is calculated as

(5) $$\left\{\begin{array}{c} \Delta x_{i}=x_{i}-x_{ci}\\ \Delta y_{i}=y_{i}-y_{ci} \end{array}\right.$$

where ($\Delta x_{i},\Delta y_{i}$) is the image shift between the real position ($x_{i},y_{i}$) and the ideal position ($x_{ci},y_{ci}$) of *i*th GCP.

#### 3.2.3. Dense GCP Image Shift Calculation

The raw images with periodic wavy patterns are selected as the measurement scenes, and a number of GCPs with intensive distribution from each measurement scene were extracted and matched with periodic wavy patterns. The sampling interval of the GCPs is set to smaller; these GCPs are defined as dense GCPs. The exact number of GCPs is determined by the sparsity and length of the recovered signal, the recovery algorithm, and so on. Generally speaking, the length of the measurements is far below the length of the recovered signal [14]. The rest of this step follows the same process as Step 2.

#### 3.2.4. Equivalent Bias Angle Recovery

The most important thing in this crucial step is the signal fusion. It is known that the compressive sensing method can only recover 1-D column vectors, therefore, 3-D equivalent bias angles signals must be merged into a 1-D time varying signal according to the time order. The problem regarding solving 3-D signal errors is transformed into the problem relating to 1-D signal recovery. After obtaining the image shift of GCPs, the remaining tasks of this step are to select a desired sparse basis and an appropriate recovery algorithm and to construct a suitable measurement matrix to exactly recover the 1-D time varying signal. The 3-D signals are separated from the 1-D recovered signal. The details of this important work are presented in the following sections.

#### 3.2.5. Image Calibration

There are two processes in this step. One involves calculating the calibrated position, and the other is to do with resampling the raw image. Once the equivalent bias angles are recovered, the calibrated image positions are easily calculated using Equation (2) with the imaging parameters. The image resampling can be completed using the bilinear interpolation method. The gray values of the calibrated image are obtained according to the calibrated image positions and the raw image.

### 3.3. Criticism of the Proposed Method

If the equivalent bias angles are considered to be errors in an ideal situation, the estimation of equivalent bias angles is a typical adjustment of indirect observations. The function model of the adjustment of these indirect observations is expressed as

(6) L=BX+d

where L is the observation, B is the measurement matrix, X is the estimated parameter, and d is an offset constant.

Generally, the estimated parameter is expressed as

(7) $$X=X_{0}+\hat{x}$$

where $X_{0}$ is the ideal value of the estimated parameter and $\hat{x}$ is the correction of the estimated parameter. 

Let

(8) $$L_{1}=L-L_{0}=L-(BX_{0}+d)$$

where $L_{0}$ is the ideal observation value.

According to Equations (6) and (7), Equation (8) can be simplified as

(9) $$L_{1}=B\hat{x}$$

where $\hat{x}$ is the equivalent bias angle, $L_{1}$ is the image shift of the GCP, and B is the measurement matrix. When there is nonlinear relation between L and X(L=F(X)), partial derivative of function F(X) must be sought to obtain the measurement matrix B.

#### 3.3.1. Measurement Matrix and Measurement Equation

(a) Measurement variables

According to Equation (8), $L_{1}$ is determined by L and $L_{0}.L$ is the real position of the GCP and $L_{0}$ is the ideal position of the GCP when M GCPs are extracted and matched from the raw image sequence. The L and $L_{0}$ are expressed as

(10) $$\begin{array}{l} L={\left[\begin{array}{ccccccc} x_{1} & y_{1} & x_{2} & y_{2} & \cdots & x_{M} & y_{M} \end{array}\right]}^{T}\\ d={\left[\begin{array}{ccccccc} x_{c1} & y_{c1} & x_{c2} & y_{c2} & \cdots & x_{cM} & y_{cM} \end{array}\right]}^{T} \end{array}$$

According to Equation (5), $L_{1}$ is expressed as

(11) $$\begin{array}{l} L_{1}={\left[\begin{array}{ccccccc} \Delta x_{1} & \Delta y_{1} & \Delta x_{2} & \Delta y_{2} & \cdots & \Delta x_{M} & \Delta y_{M} \end{array}\right]}^{T}\\ ={\left[\begin{array}{ccccccc} x_{1}-x_{c1} & y_{1}-y_{c1} & x_{2}-x_{c2} & y_{2}-y_{c2} & \cdots & x_{M}-x_{cM} & y_{M}-y_{cM} \end{array}\right]}^{T} \end{array}$$

where $L_{1}$ is a 2M×1 vector.

(b) Measurement equation

According to Equation (9), the measurement equation of one GCP is expressed as

(12) $${\left[\begin{array}{cc} \Delta x_{m} & \Delta y_{m} \end{array}\right]}^{T}=B_{mn}{\left[\begin{array}{ccc} \alpha_{n} & \beta_{n} & \theta_{n} \end{array}\right]}^{T}$$

where ${\left[\begin{array}{ccc} \alpha_{n} & \beta_{n} & \theta_{n} \end{array}\right]}^{T}$ are the equivalent bias angles of the mth GCP and $B_{mn}$ is the 2×3 measurement matrix. 

The measurement equation of M GCP is expressed as

(13) $$L_{1}=B\hat{x}\;\;\Rightarrow \;\left[\begin{array}{c} \Delta x_{1}\\ \Delta y_{1}\\ \Delta x_{2}\\ \Delta y_{2}\\ \vdots \\ \Delta x_{M}\\ \Delta y_{M} \end{array}\right]=\left[\begin{array}{cccc} B_{11} & 0 & \cdots & 0\\ 0 & B_{22} & \cdots & 0\\ \vdots & \vdots & \ddots & \vdots \\ 0 & 0 & \cdots & B_{MN} \end{array}\right]\left[\begin{array}{c} \alpha_{1}\\ \beta_{1}\\ \theta_{1}\\ \alpha_{2}\\ \beta_{2}\\ \theta_{2}\\ \vdots \\ \alpha_{N}\\ \beta_{N}\\ \theta_{N} \end{array}\right]=\left[\begin{array}{cccccccccc} b_{11}^{1} & b_{12}^{1} & b_{13}^{1} & 0 & 0 & 0 & \cdots & 0 & 0 & 0\\ b_{21}^{1} & b_{22}^{1} & b_{23}^{1} & 0 & 0 & 0 & \cdots & 0 & 0 & 0\\ 0 & 0 & 0 & b_{11}^{2} & b_{12}^{2} & b_{13}^{2} & \cdots & 0 & 0 & 0\\ 0 & 0 & 0 & b_{21}^{2} & b_{21}^{2} & b_{21}^{2} & \cdots & 0 & 0 & 0\\ \vdots & \vdots & \vdots & \vdots & \vdots & \vdots & \ddots & \vdots & \vdots & \vdots \\ 0 & 0 & 0 & 0 & 0 & 0 & \cdots & b_{11}^{M} & b_{12}^{M} & b_{13}^{M}\\ 0 & 0 & 0 & 0 & 0 & 0 & \cdots & b_{21}^{M} & b_{22}^{M} & b_{23}^{M} \end{array}\right]\left[\begin{array}{c} \alpha_{1}\\ \beta_{1}\\ \theta_{1}\\ \alpha_{2}\\ \beta_{2}\\ \theta_{2}\\ \vdots \\ \alpha_{N}\\ \beta_{N}\\ \theta_{N} \end{array}\right]$$

where $L_{1}$ is a 2M×1 measurement vector, B is a 2M×3N measurement matrix, and $\hat{x}$ is a 3N×1 merged equivalent bias angle vector.

(c) Linearization of measurement equation

The other important issue regards obtaining the element of the measurement matrix B. To reduce the complexity of the algorithm, the linearization of measurement equations for each GCP must be completed before recovering the signal. According to Equation (2), the relationship between the estimated parameters and the measurements is nonlinear, therefore a partial derivative of Equation (2) should be sought to obtain the measurement matrix B. However, seeking a partial derivative of Equation (2) is difficult, and the numerical analysis method is used to estimate the elements of the measurement matrix B. Next, the elements $b_{11}^{m}$ and $b_{21}^{m}$ of $B_{mn}$ are taken as examples to introduce the details of the solution.

1. The upper limit of the equivalent bias angles A, the increment Δγ in each step, and the cycle index I=AΔγ are determined;
2. The first parameter $\alpha_{n}$ of nth equivalent bias angles is set to i×Δγ       i∈[0,I] and the other parameters are set to zero;
3. The image positions of the mth GCP by using the imaging parameters and the equivalent bias angles $\left(\begin{array}{ccc} i\times \Delta \gamma & 0 & 0 \end{array}\right)\;\;\;\;\;i\in [0,I]$ are calculated’
4. After obtaining the I+1 image positions $\left(\begin{array}{cc} x_{0} & y_{0} \end{array}\right)$,$\left(\begin{array}{cc} x_{1} & y_{1} \end{array}\right)$, and $\left(\begin{array}{cc} x_{I} & y_{I} \end{array}\right)$, the variations of image positions $\Delta x_{i}$ and $\Delta y_{i}$ are calculated using
  (14) $$\left\{\begin{array}{c} \Delta x_{i}=x_{i}-x_{0}\\ \Delta y_{i}=y_{i}-y_{0} \end{array}\right.\;\;\;\;\;\;\;\;\;\;i\in [1,I]$$
5. $b_{11}^{m}$ and $b_{21}^{m}$ are calculated as follows. The other elements of $B_{mn}$ can be calculated in the same way
  (15) $$\left\{\begin{array}{c} b_{11}^{m}=\frac{1}{I}\sum_{i=1}^{I}\frac{\Delta x_{i}}{i\times \Delta \gamma }\\ b_{21}^{m}=\frac{1}{I}\sum_{i=1}^{I}\frac{\Delta y_{i}}{i\times \Delta \gamma } \end{array}\right..$$

#### 3.3.2. Sparse Basis and Sparse Representation

**Figure 3:**
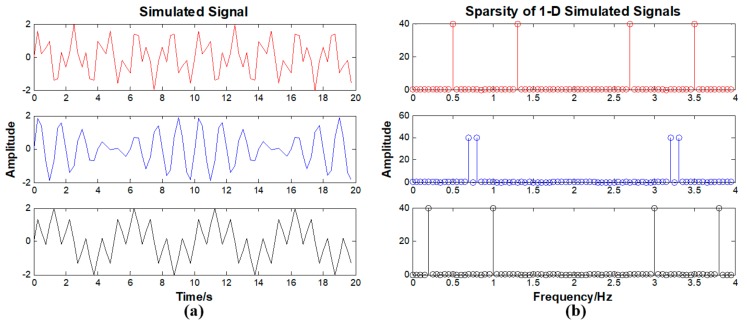
(**a**) Simulated 3-D equivalent bias angles signals. (**b**) Results of 1-D DFT for the 1-D equivalent bias angle signal.

The equivalent bias angles signals based on Equation (4) are not sparse in the time domain. According to previous studies [8,17], the DFT converts equivalent bias angles signals into the sparse representation. The simulated signals based on Equation (4) are displayed in Figure 3a. The three curves in Figure 3a represent the simulated three-dimensional equivalent bias angles signals with different constant errors and period components. The red curve indicates the X direction, the blue curve indicates the Y direction, and the black curve indicates the Z direction. The results of the 1-D DFT are displayed in Figure 3b.

**Figure 4:**
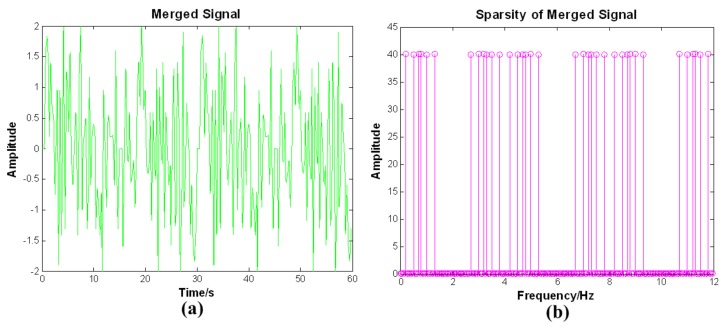
(**a**) 1-D merged signal. (**b**) Results of the 1-D DFT.

To easily recover the equivalent bias angles signals, the 3-D equivalent bias angle signals need to be merged into a 1-D signal according to time order. The 1-D merged signal and the results of the 1-D DFT are displayed in Figure 4.

From Figure 3b, it is seen that the 3-D equivalent bias angle signals are sparse in the frequency domain and the 1-D merged signal is sparse in the frequency domain, as seen in Figure 4b. Therefore, we select the 1-D DFT basis as the sparse basis Ψ in the proposed method. The performances of the other sparse bases are all worse than the 1-D DFT basis. The definition of sparse basis Ψ is

(16) $$\Psi =\frac{1}{N}\left[\begin{array}{cccc} e^{j2\pi (0\times 0)/N} & e^{j2\pi (1\times 0)/N} & \cdots & e^{j2\pi ((N-1)\times 0)/N}\\ e^{j2\pi (0\times 1)/N} & e^{j2\pi (1\times 1)/N} & \cdots & e^{j2\pi ((N-1)\times 1)/N}\\ \vdots & \vdots & \ddots & \vdots \\ e^{j2\pi (0\times (N-1))/N} & e^{j2\pi (1\times (N-1))/N} & \cdots & e^{j2\pi ((N-1)\times (N-1))/N} \end{array}\right]$$

#### 3.3.3. Signal Recovery

It is obvious from Equation (12) that the 2-D measurement equation used to solve the 3-D equivalent bias angles of one GCP is a pathological problem. The traditional methods assume that the equivalent bias angles are unchanged in the frame period and use classical optimal estimation methods—such as least squares estimation, Bayes estimation, or maximum likelihood estimation—to combine a number of GCPs to estimate the equivalent bias angles of each frame. However, this assumption is unreasonable when equivalent bias angles contain short-period errors. Due to M<<N, the solution of Equation (13) is highly undetermined; therefore, the traditional methods do not work in this situation. Fortunately, it is possible to exactly recover the values of $\hat{x}$ using compressive sensing if $\hat{x}$ is represented by the sparse basis.

The measurement equation of M GCP in Equation (13) is rewritten as

(17) $$L_{1}=B\hat{x}=B\Psi f=\tilde{B}f$$

where Ψ is a 3N×3N sparse basis, f is a 3N×1 sparse representation, $\hat{x}=\Psi f$, and $\tilde{B}=B\Psi$ is a 2M×3N sensing matrix. In Equation (17), $L_{1}$ is considered to be the product of f times $\tilde{B}$ [13]. The 1-D DFT method achieves RIP [17]. The GCP is selected randomly when the measurement matrix B achieves RIP, therefore, the sensing matrix $\tilde{B}$ also achieves RIP. We use the matching pursuit algorithm to recover the exact values of f due to its lower computational complexity [18,19]. The merged equivalent bias angle signal $\hat{x}$ is obtained as

(18) $$\hat{x}=\Psi f$$

## 4. Experiments and Analysis

Two experiments were used to compare the proposed method with the RFM method in this section. The first method employs the image data from the Hyperion of Earth Observing 1 (EO-1) and shows the performances of two methods designed to estimate the long-period components of equivalent bias angles. The second method, which uses the image data from the Panchromatic Remote-sensing Instrument for Stereo Mapping (PRISM) of the Advanced Land Observing Satellite (ALOS), shows that the proposed method has a superior performance regarding the estimation of the short-period components. The details of the experiments are described as follows.

### 4.1. Hyperion Data Experiment

#### 4.1.1. Data Description

**Figure 5:**
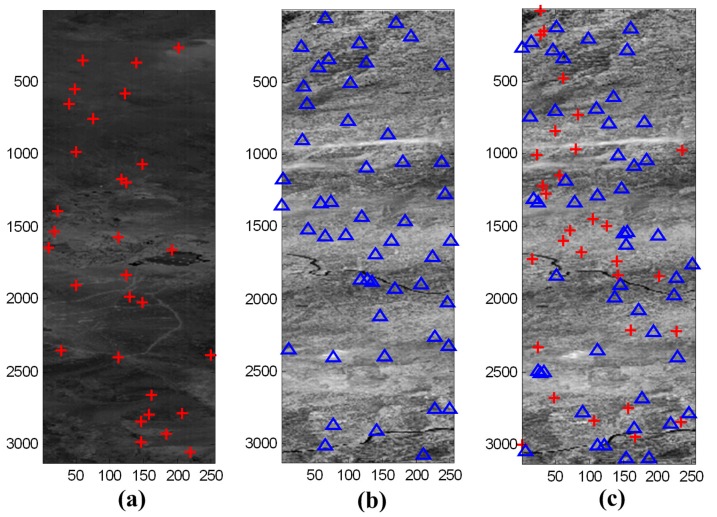
(**a**) Measurement scene and (**b**) verification scene of the proposed method. (**c**) Measurement and verification scene of the rational functional model (RFM) method (30 GCPs and 50 random check points (RCPs)).

Hyperion, as the main payload of EO-1, is a typical linear array push-broom sensor. A total of 100 Hyperion frame images from 7 June 2002 were selected as the experimental data. In the proposed method, we selected 40 frames with some evenly distributed GCPs from the experimental data as the measurement scenes. The measurement scene used in the proposed method is represented in Figure 5a, with the red crosses denoting the GCPs. The rest of the frames were selected as verification scenes, which evenly distribute 50 random check points (RCPs) in each scene. One verification scene used in the proposed method is represented in Figure 5b, with the blue triangles denoting the RCPs. For the RFM method, we extracted some GCPs in each frame; the verification scenes were the same as in the proposed method. The measurement and verification scene of the RFM method are represented in Figure 5c. The GCPs of the measurement scenes were used as the inputs for both methods. The RCPs in the verification scenes were selected as the valuators to exactly evaluate the calibration performances of both methods. To adequately compare the methods, we designed 10 testing cases with different numbers of GCPs (5–50) and counted the calibration results. GCPs appeared to distribute evenly in this experiment. The original size (3129×256) of the Hyperion images was changed in order to be presented in Figure 5.

#### 4.1.2. Experimental Results

**Figure 6:**
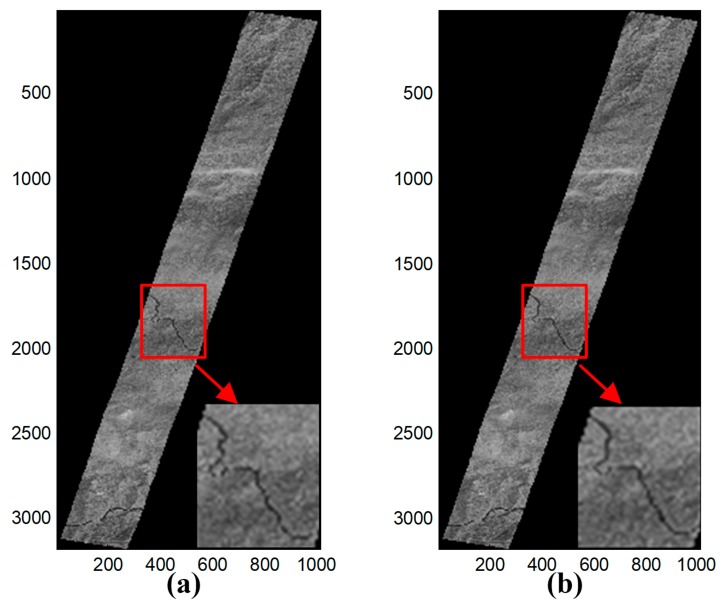
The calibration results of (**a**) the proposed method and (**b**) the RFM method.

The calibration results of both methods for one frame showing 30 GCPs are displayed in Figure 6. The calibration results of the two methods in this case could be distinguished by the naked eye, even when details were closely examined (Figure 6).

**Figure 7:**
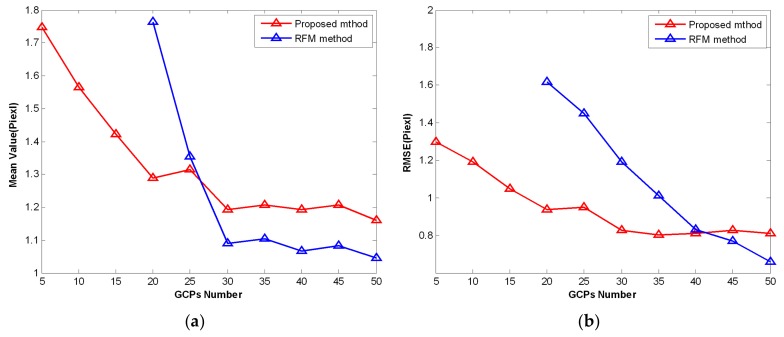
The variation trends of (**a**) mean value and (**b**) root mean square error (RMSE) of the number of GCPs across the two methods.

To make accurate comparisons between the two methods, the calibrated residuals of the RCPs were employed in the statistical analysis. The mean value and root mean square error (RMSE) of the calibrated residuals were used as the measurable indicators. The variation trends of the mean value and the RMSE for the total GCPs across the two methods are shown in Figure 7. At least 19 GCPs were required to solve the coefficients of RFM; the RFM method did not work with 5, 10, or 15 GCPs. As the number of GCPs increased, the mean value of the two methods gradually decreased, as shown in Figure 7a. Similarly, the RMSE of the two methods gradually decreased in Figure 7b as the number of GCPs increased, i.e., the calibration performance of the two methods improved when the number of GCPs increased. The RFM method was superior in calibration performance when presented with enough GCPs. However, the proposed method did well when insufficient GCPs were available. Therefore, the proposed method achieved a better calibration performance in situations with insufficient GCPs compared with the RFM method.

### 4.2. Experiment on ALOS Data

#### 4.2.1. Data Description

**Figure 8:**
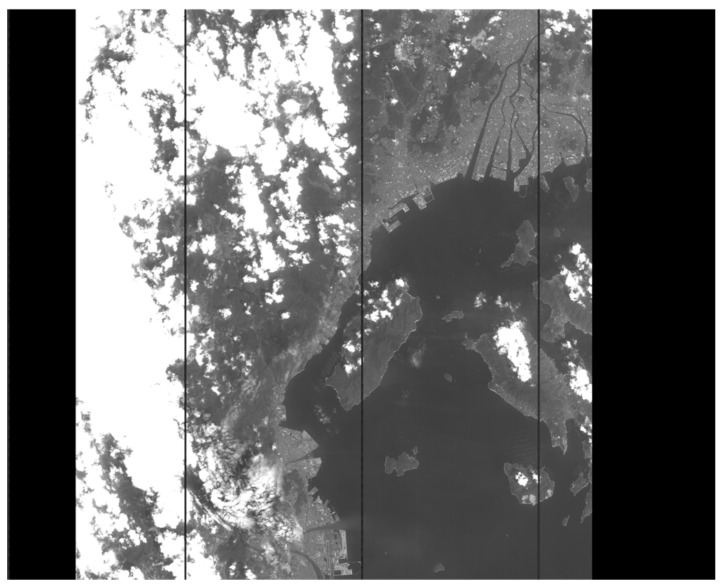
Image data of a typical scene.

PRISM, the primary sensor carried by ALOS, was designed to obtain a high-resolution digital surface model to derive orthoimages on a global scale. PRISM has three panchromatic linear array push-broom sensors (forward, nadir, and backward) with a 2.5 m spatial resolution and a width of 35 km swaths. The raw image data of PRISM was observed on 20 August 2006 and covered 50 scenes; these were used as the experimental data. To verify the performances of the two methods regarding the estimation of short-period components, one scene from the experimental data was used as the typical scene, as shown in Figure 8. We extracted 100 evenly distributed GCPs as inputs for the two methods and 50 evenly distributed RCPs to evaluate the calibration performances of the two methods in this typical scene. Also, we randomly chose 20 scenes with 30 evenly distributed GCPs as the measurement data from the rest of the experimental data. 

#### 4.2.2. Experimental Results

**Figure 9:**
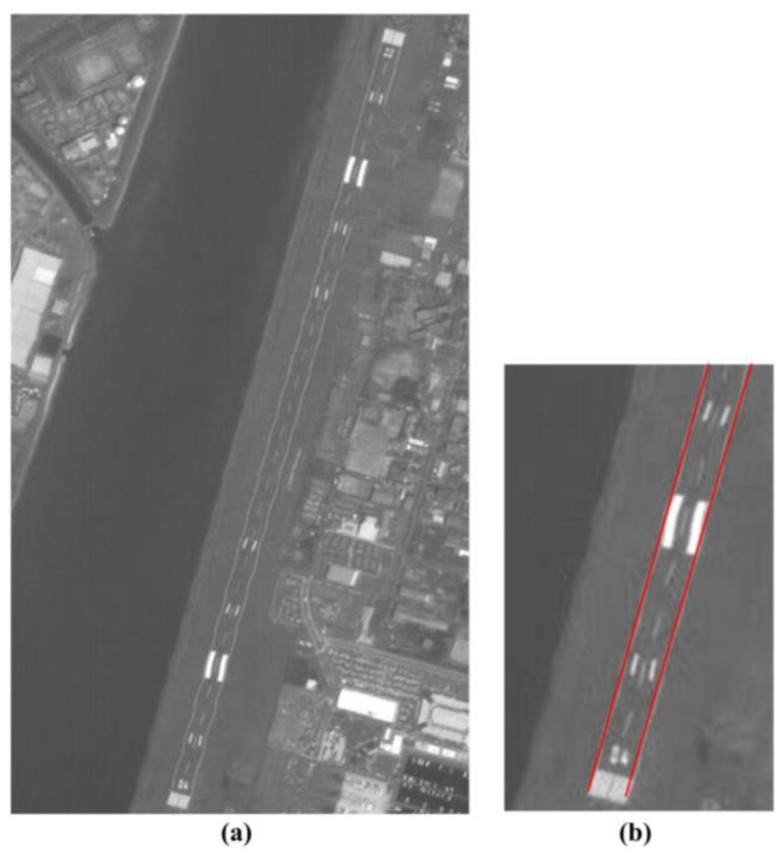
The line detection results of a typical scene. (**a**) Hiroshima West Airfield and (**b**) enlarged details.

We used the line or edge detection algorithm to extract the road in a typical scene; the detection results are shown in Figure 9.

**Figure 10:**
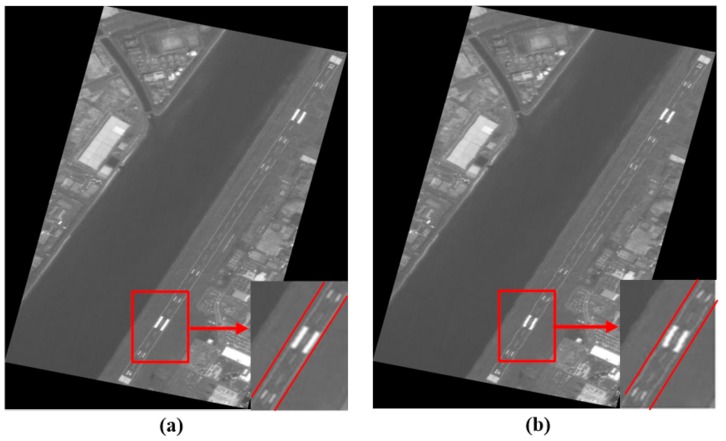
Partial details of the calibration results for a typical scene. (**a**) Proposed method and (**b**) RFM method.

Figure 9a shows the Hiroshima West Airfield. As seen in Figure 9b, the airfield runway had obvious periodic wavy patterns which were caused by short-period errors. The optical system of PRISM was affected by the motion of another sensor’s mirror when this image was in the process of being captured, therefore, Step 3 of the proposed method was performed to deal with this image data. The partial details of the calibration results of a typical scene using the different methods are shown in Figure 10. The periodic wavy patterns in the calibration results of the proposed method are hardly recognizable in Figure 10a, but the periodic wavy patterns are easily recognized in the calibration results of the RFM method in Figure 10b.

The calibration results of the two methods for 50 RCPs in typical scene are shown in Table 1, which shows that the mean value and RMSE of the calibrated results for the proposed method were less than those of the RFM method. The results of this experiment indicated that the proposed method did well in estimating the short-period components of equivalent bias angles when compared with the RFM method.

## 5. Conclusions

Geometric calibration must be carried out before the application of raw images. This paper proposed a geometric calibration method using sparse recovery to remove linear array push-broom sensor bias. The errors in the imaging process were approximated to the equivalent bias angles in this method. By using the sparse recovery method, the proposed method exactly estimated long-period errors with a small number of GCPs available. Also, the proposed method effectively removed short-period errors by recognizing periodic wavy patterns in advance. The preliminary experimental results indicated the practicality and superior calibration performance of the proposed method when used for image data captured by the EO-1 and ALOS satellites. Compared with the traditional methods, the proposed method did well in situations with insufficient GCPs and short-period error calibration.

Future research will focus on the effects of GCP distribution on the proposed method. It is also important to apply the proposed method to other types of sensors.

## Figures and Tables

**Table 1:** Calibration results of the two methods using 50 RCPs in a typical scene.

	**Mean Value** **(urad)**	**RMSE** **(Pixels)**
Proposed Method	0.43	0.68
RFM Method	1.67	2.03
